# *Stenotrophomonas maltophilia* Epidemiology, Resistance Characteristics, and Clinical Outcomes: Understanding of the Recent Three Years’ Trends

**DOI:** 10.3390/microorganisms10122506

**Published:** 2022-12-18

**Authors:** Taghreed A. Hafiz, Esraa Aldawood, Alaa Albloshi, Shahad S. Alghamdi, Murad A. Mubaraki, Ahmed S. Alyami, Marwh G. Aldriwesh

**Affiliations:** 1 Clinical Laboratory Sciences Department, College of Applied Medical Sciences, King Saud University, Riyadh 12372, Saudi Arabia; 2 Regional Laboratory and Central Blood Bank, Microbiology Department, Albaha 65715, Saudi Arabia; 3 Pathology and Clinical Laboratory Medicine, King Fahad Medical City, Riyadh 11525, Saudi Arabia; 4 Department of Clinical Laboratory Sciences, College of Applied Medical Sciences, King Saud bin Abdulaziz University for Health Sciences, Riyadh 11481, Saudi Arabia; 5 King Abdullah International Medical Research Center, Riyadh 11481, Saudi Arabia

**Keywords:** *Stenotrophomonas maltophilia*, respiratory, ICU, nosocomial infection, MDR, PDR, COVID-19

## Abstract

Background. *Stenotrophomonas maltophilia* is an emerging pathogen classified as a public health concern, that infects critically ill patients and has expressed resistance against antimicrobial therapy. The aim of this study was to examine the epidemiological pattern, resistance characteristics and clinical outcomes of *S. maltophilia* infections in hospitalized patients. Methods. The study included 393 *S. maltophilia* isolates from different clinical specimens as well as the clinical data of 209 Intensive Care Unit (ICU) patients. The patients’ data were obtained from medical and laboratory files. Descriptive statistics and a univariate analysis were used to report and compare the demographics, clinical data, and outcomes. Results. The *S. maltophilia* was mostly isolated from the respiratory specimens of ICU patients. The adult patients were more likely to develop serious infections and worse outcomes than were pediatric patients. The most common co-infecting pathogens were SARS-CoV2 and *Pseudomonas aeruginosa*. The death rate was 44.5% and increased to 47.1% in the case of a respiratory infection. Septic shock was the most significant predictor of mortality. Older age and mechanical ventilation were independent and significant risk factors that worsened the outcomes in patients with respiratory infections. Conclusions. The identification of *S. maltophilia* as a threat highlights the importance of surveillance studies in this region.

## 1. Introduction

*Stenotrophomonas maltophilia* (*S. maltophilia*) is a nonfermenting gram-negative bacillus bacterium [1]. It can be found in different sources of the environment including soil, plants, and animals, as well as it can be detected in an aquatic environment [2]. It is known that *S. maltophilia* is the only species of the *Stenotrophomonas* genus that can cause infections in humans, mainly among immunocompromised patients and hospitalized patients, and specifically among Intensive Care Unit (ICU) patients who have undergone prosthetic devices such as catheters, mechanical ventilators, and feeding tubes, as it is capable of producing a biofilm on these surfaces [3]. However, it is uncommon for *S. maltophilia* to cause community-acquired infections [4]. *S. maltophilia* is considered to be the most prevalent organism isolated in clinical laboratories after *Pseudomonas aeruginosa*, *Acinetobacter* spp., and the *Burkholderia cepacia* complex [5]. Most of the infections caused by *S. maltophilia* occur in the lower respiratory tract in the form of tracheobronchitis or pneumonia because of mechanical ventilation, in addition to blood infections such as bacteremia [6]. There are other infections that can be caused by *S. maltophilia*, such as wound and soft tissue infections, meningitis, peritonitis, urinary tract infections (UTI), and bone/ joint infections [4]. The mortality rate of an *S. maltophilia* infection is significantly high, as it reaches 75% when associated with pneumonia and 20% when associated with bacteremia [7]. *S. maltophilia* is identified to be resistant to different types of antimicrobials via different mechanisms, including the production of aminoglycoside acetyltransferase and enzymes, which immobilize erythromycin. In addition, *S. maltophilia* can possess certain genes that encode efflux pumps [8]. The resistance can be intrinsic or acquired by either mutations or through carrying the resistant genes via a horizontal gene transfer [2]. *S. maltophilia has multi-drug resistance (MDR) to certain types of antimicrobials; additionally, pan-resistant strains of S. maltophilia have been found in hospitals as a result of inappropriate antibiotic use, particularly of broad-spectrum antibiotics* [8]. The therapeutic options for treating *S. maltophilia* infections are limited due to its resistance to a wide range of antibiotics such as aminoglycoside and a variety of ß-lactam antibiotics including carbapenem [9]. In addition to the outbreak caused by the virus severe acute respiratory syndrome coronavirus 2 (SARS-CoV2), which was initiated in Wuhan at the end of 2019, there is an elevation in the prevalence of multi-drug resistance (MDR) bacteria including *S. maltophilia*. This elevation in the resistance occurs as a consequence of the increased utilization of anti-microbial agents among hospitalized COVID-19 patients [10]. However, there are few studies concerned with investigating the resistance pattern of *S. maltophilia* before and during the outbreak of SARS-CoV-2. The aim of this study was therefore to investigate the epidemiological pattern, resistance characteristics, and clinical outcomes of *S. maltophilia* in depth over the last three years in Saudi Arabia.

## 2. Materials and Methods

### 2.1. Study Design and Setting

A retrospective study was conducted over three years from January 2019 to December 2021 at King Fahad Medical City (KFMC), with a capacity of 1200 beds, in Riyadh, Saudi Arabia. A total of 393 *S. maltophilia* isolates from various clinical samples were analyzed. The clinical histories of 209 ICU patients were also included in this study.

### 2.2. Data Collection

A total of 393 samples of *S. maltophilia* were collected from various sources, which included blood (central and peripheral lines), respiratory (sputum and endotracheal), urine (mid-stream urine, indwelling catheter, and in and out catheter), and miscellaneous (abscess, wound, tissue, body fluid, and device) sources. The inclusion criteria were: (A) Age was divided into pediatric and adult categories. The pediatric category included three sub-categories: infant (age 1 Y); children (age from 1 to 10 Y); and adolescent (age from 11 to 18 Y). The adults were divided into four groups: group one (from 19 to 44 Y), group two (from 45 to 64 Y), group three (from 65 to 84 Y), and group four (age 85 and above). (B) Ward or clinic that the patient was admitted to, including emergency, ICU, ward, and outpatient clinic. (C) Sample source and location/site. (D) Bacterial resistant category. Any growth other than *S. maltophilia* was excluded from the data. The clinical histories were collected from the KFMC databases for patients admitted to the ICU, including pediatric and adult patients. The clinical histories collected for the ICU patients included different criteria, which were: (1) co-infection, if present or not; (2) exposure to carbapenem or other antibiotic in the past 14 to 30 days; (3) renal dialysis at isolation or not; (4) on mechanical ventilation or not; (5) chronic disease such as DM, hypertension, renal disease, or malignancy; (6) the presence of clinical symptoms such as fever, GIT symptoms, or respiratory symptoms; (7) the presence of a wound or urinary tract infection; (8) the presence of bacteremia or septicemia; (9) clinical outcomes for the patient and additional notes if present.

### 2.3. S. maltophilia Identification and Antimicrobial Susceptibility Testing

All of the isolates were presumptively identified as Stenotrophomonas species by the aid of a Phoenix BD instrument for full identification and sensitivity testing. Only patients whose isolates were definitively identified as *S. maltophilia* were included. Antimicrobial sensitivity testing (AST) was done for the following antibiotics: ceftazidime (CTZ), levofloxacin (LVX), and trimethoprim-sulfamethoxazole (TMP-SMX). The results were interpreted and reported according to the 32nd Edition of the CLSI-M100 document and classified as susceptible (S), intermediate (I), and resistant (R). The confirmation of resistant isolates was performed by an E-test. The *S. maltophilia* isolates were categorized based on their resistance to antibiotics, according to International Consensus [11].

### 2.4. Statistical Analysis

All data were analyzed using a GraphPad prism statistical project version 9.3.1. The demographic characteristics of the entire study population with an *S. maltophilia* infection were summarized. A univariate analysis to compare clinical characteristics between adults and pediatric ICU-admitted patients was obtained using Fisher’s exact test, with a *p*-value of less than 0.05 being statistically significant. Similarly, a multivariate analysis was used to compare the outcomes and risk factors linked to death among ICU patients. Relative risk (RR) was calculated to indicate how much the risk factors increased the risk of death among those patients with a respiratory infection, and the results were reported as RR and 95% confidence interval (CI). Additionally, the AMT was presented in percentages.

### 2.5. Ethical Approval

The project was approved by the local ethical research committee of King Fahad Medical City. Consent was obtained from KFMC according to ICH GCP guidelines with the ethical code of IRB log number: 21-426E.

## 3. Results

### 3.1. Demographic Characteristics of Patients with S. maltophilia Infection

A total of 393 isolates of *S. maltophilia* were isolated from different clinical specimens including the sputum, endotracheal secretions, the urine, the blood, and other miscellaneous body fluids. The majority of the isolates were obtained from respiratory specimens (65.4%), followed by blood cultures (17.3%). Approximately half of the *S. maltophilia* isolates (53.2%) mainly originated from the ICU (209/393). The demographic characteristics of patients are summarized in Table 1.

### 3.2. Comparison of Clinical Characteristics among Adult and Pediatric ICU Patients

A univariant analysis was conducted to compare the clinical characteristics between adult and pediatric ICU patients (*n* = 209). The adult patients infected with *S. maltophilia* were significantly more likely to develop a co-infection, a urinary tract infection, septic shock, and death than were pediatric patients. Almost half of the ICU patients were co-infected with another organism (*n* = 106; 50.7%). The co-infection was mostly caused by gram-negative bacteria (64%; *Pseudomonas aeruginosa, Klebsiella pneumoniae, Klebsiella oxytoca, Acinetobacter baumannii, Enterobacter cloacae, Escherichia coli, Serratia species, and Proteus mirabilis*), followed by a SARS-CoV-2 infection (48%). The most frequent co-isolated species were *P. aeruginosa* (28%), *K. pneumoniae* (12%), and *A. baumannii* (9%). Co-infections with the influenza A virus, fungi, and gram-positive bacteria (*Staphylococcus aureus, Staphylococcus epidermidis*, and *Enterococcus faecium*) were also detected. In addition, there was a significant association between adults aged more than 18 and some clinical characteristics including fever, respiratory symptoms, mechanical ventilation, recent antibiotic pre-exposure, and chronic diseases (Table 2). Diabetes mellitus and hypertension were the most significant chronic illnesses associated with *S. maltophilia* infections among adult ICU patients.

### 3.3. Clinical Outcomes and Factors Associated with Mortality of ICU Patients with S. maltophilia Infection

The overall mortality rate of an *S. maltophilia* infection among ICU patients was 44.5% and a respiratory infection increased the mortality rate to 47.1%. To evaluate the risk factors associated with mortality between the resolved and the deceased patients, univariant analysis was conducted (Table 3). Several factors were tested for their potential associations with mortality among the infected ICU patients with *S. maltophilia*: age above 18 years, a positive respiratory culture, septicemia, septic shock, malignancy, prior exposure to antimicrobials, and the use of mechanical ventilation. Upon univariate analysis, there were three factors significantly associated with mortality among the infected ICU patients with *S. maltophilia*: age above 18 years, septicemia, and septic shock at *p* = 0.0049, *p* = 0.0321, and *p* < 0.0001, respectively. However, septic shock was the leading cause of death in *S. maltophilia* infected ICU patients (*p* < 0.0001).

There was a considerable percentage (82%) of ICU patients who had an *S. maltophilia* respiratory infection. Therefore, a multivariate analysis was conducted to identify the significant predictors for mortality among those patients (Table 4). The results of the multivariate analysis indicated that only the age above 18 years (RR = 1.264; 95% CI: 1.036–1.557; *p* = 0.031) and the use of mechanical ventilation (RR = 1.138; 95% CI: 1.020–1.290; *p* = 0.034) were independent and significant risk factors for mortality among the infected ICU patients with respiratory *S. maltophilia* infections as shown in Table 4. In other words, the risk of mortality was increased by 26% in patients aged above 18 years. Furthermore, the risk of death was increased by 14% among mechanically ventilated patients relative to non-ventilated ICU patients.

### 3.4. Antibiotic Susceptibility of S. maltophilia Isolates

**Figure 1:**
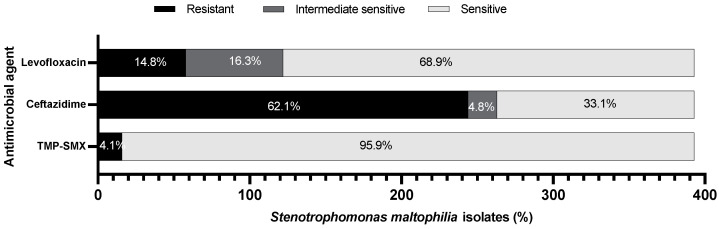
Antibiotic susceptibility of *S. maltophilia* isolates (*n* = 393).

The antimicrobial susceptibility testing (AST) of *S. maltophilia* isolates from the 393 patients is illustrated in Figure 1. *S. maltophilia* susceptibility to TMP/SMX was the highest (95.9%), followed by levofloxacin (68.9%) and ceftazidime (33.1%). Therefore, the isolates showed higher resistance to ceftazidime (62.1%) than to levofloxacin (14.8%) and TMP/SMX (4.1%).

### 3.5. S. maltophilia Infection before and during COVID-19 Pandemic

The trends of *S. maltophilia* infections before and during the COVID-19 pandemic were investigated among ICU and non-ICU patients. As detailed in Table 5, before and during the COVID-19 pandemic, the frequency of positive respiratory cultures isolated from ICU patients was consistently higher than non-ICU patients (*p* < 0.0001) over a three year period (2019, 2020, and 2021). On the contrary, the number of *S. maltophilia* isolates from non-respiratory specimens was higher among non-ICU patients compared to ICU patients before and during the COVID-19 pandemic. Furthermore, the frequency of bloodstream infections increased consistently, especially among non-ICU patients, at 17.7%, 27.9%, and 31.2% in 2019, 2020, and 2021, respectively.

## 4. Discussion

*S. maltophilia* is commonly circulated in natural environments such as water and soil. This bacterium can also be present in hospital environments, which may result in nosocomial infections. Unfortunately, the detection of *S. maltophilia* in ICUs has increased over the past few years, which has raised concerns that urge an in-depth study of the risk factors associated with this pathogen in ICUs. To our knowledge, there were no studies performed in Saudi Arabia concerning *S. maltophilia* clinical isolates before and during the COVID-19 era, and no studies have considered both adult and pediatric patients.

Our study included 393 isolates of *S. maltophilia* collected from January 2019 to December 2021 at KFMC. The isolates were collected from different sites, but most were from respiratory, followed by blood, and more than half of the specimens were obtained from ICU patients. These findings are not surprising, as a study that was performed in KKUH in Riyadh (2012) also reported that *S. maltophilia* mainly causes respiratory infections and is predominantly isolated from ICU patients [12].

Our study revealed that the numbers of *S. maltophilia* isolates before and during the COVID-19 pandemic have not changed significantly in both the ICU and non-ICU patients (Table 5). One reason for this is that during the pandemic, most of the non-urgent surgeries were canceled, therefore the number of patients admitted was reduced to allow beds to be available for COVID-19 cases. Moreover, the infection control measures that were taken in hospitals during the COVID-19 era may affect the rate of nosocomial infections caused by *S. maltophilia.* Our study showed that the severity of *S. maltophilia* and the risk of developing co-infections is more in adult patients compared with pediatric patients. To our knowledge, there were no previous studies that compared adults to children in terms of severity; most studies of *S. maltophilia* in Saudi Arabia focused on pediatrics [13,14].

The results presented herein indicate that respiratory infections of *S. maltophilia* are the highest among other types of infections (Table 5), which is consistent with what was reported in the previous study [15]. This might be due to the ability of *S. maltophilia* to form biofilms and colonize on the respiratory tracts of hospitalized patients [16]. The observed higher number of respiratory infections among ICU patients might be reflected using mechanical ventilation, which was reported previously as a risk factor for the development of an *S. maltophilia* respiratory infection [15].

The most common co-infections with *S. maltophilia* were found in this study to be gram-negative bacterial infections. Similarly, various studies have reported that the polymicrobial infection associated with *S. maltophilia* was caused by gram-negative bacteria [14,17]. Additionally, a recent study has reported the presence of *S. maltophilia* as a secondary bacterial infection in ICU-COVID-19 patients [18]. We found some clinical characteristics that were more significantly associated with adult ICU patients than pediatric ICU patients, such as fever, septic shock, and underlying conditions such as hypertension and diabetes mellitus. Our study reported the mortality rate among *S. maltophilia* ICU patients to be 44.4%, which is consistent with a study performed in KKUH in Riyadh [12]. Septic shock was the most significant predictor of mortality, which is in line with other studies that investigated *S. maltophilia* mortality risk factors [6], followed by septicemia, and then being above 18 years.

The relative risk of mortality among ICU patients with respiratory infections was investigated. Being on a mechanical ventilator and being over 18 years increased the risk of mortality by 14% and 26%, respectively. A recent metanalytic study has also identified a strong correlation between mechanical ventilators and *S. maltophilia* pneumonia in ICU patients [19]. Therefore, physicians are required to firmly follow the indications for invasive procedures and minimize unnecessary invasive procedures. Understanding the risk factors associated with *S. maltophilia* infections in the ICU and early targeted empirical treatment is crucial to reducing the mortality from *S. maltophilia*. The treatment of *S. maltophilia* is difficult, as this pathogen demonstrates high levels of intrinsic or acquired resistance to different antimicrobial agents, limiting the available options for treatment [6]. Our study showed that *S. maltophilia* isolates were mostly resistant to ceftazidime (62.1%), followed by levofloxacin (14.8%), and then TMP/SMX (4.1%). According to the study performed in KKUH in 2012, *S. maltophilia* isolates resistant to ceftazidime were (57.21%) and those resistant to TMP/SMX were (9.45%). These data indicate that ceftazidime resistance has increased, which is probably due to the extensive use of the antibiotic over the past few years, especially during the pandemic. Fortunately, TMP/SMX remained effective as empirical therapy for *S. maltophilia* infections, but resistance to this drug must be continuously monitored.

The study’s sample size included a range of diverse sample sources from various sites that identified the types of infections, which helped to clarify the sources of the *S. maltophilia* infections. Additionally, the large sample size from the ICU patients allowed us to draw very exact conclusions from the statistical analysis. However, the study’s main drawback is that it only used data from a single tertiary institution. A large-scale multicenter effective surveillance system in Riyadh and other cities in Saudi Arabia with a larger sample size is needed for a better understanding of the *S. maltophilia* threat.

## 5. Conclusions

*S. maltophilia* is an emerging pathogen causing a relatively high mortality rate in ICU patients. In the cases of respiratory infections, the fatality rate climbed to 47.1%. Septic shock was the most important predictor of death. In patients with respiratory infections, older age and mechanical ventilation were independent and substantial risk factors. Knowing the risk factors, whether they are related to the host and/or medical factors associated with *S. maltophilia* mortality, is the key to the monitoring, prevention, and control of the infections. This study will help physicians in assessing the risks of *S. maltophilia* infections in ICU patients and will enhance the management of high-risk groups.

## Figures and Tables

**Table 1:** Demographic characteristics of patients with *S. maltophilia* infections.

Characteristic	Patients, *n* = 393
Gender, *n* (%)	
Male	231 (58.8)
Female	162 (41.2)
Age group, *n* (%)	
Infants < 1 y	59 (15)
Children 1–10 y	40 (10.2)
Adolescents 11–18 y	15 (3.8)
Adults 19–44 y	65 (16.5)
Adults 45–64 y	95 (24.2)
Adults 65–84 y	101 (25.7)
Adults > 85 y	18 (4.6)
Specimen source, *n* (%)	
Respiratory	257 (65.4)
Blood	68 (17.3)
Urine	35 (8.9)
Miscellaneous ^1^	33 (8.4)
Type of ward/clinic (%)	
ICU ^2^	209 (53.2)
ER ^3^	34 (8.7)
OPC ^4^	23 (5.8)
Wards	127 (32.3)

Note: Data are presented as number of patients (*n*) with the corresponding percentage in parentheses (%). ^1^ Miscellaneous samples were abscess, wound, tissue, body fluid, and device; ^2^ ICU, Intensive Care Unit; ^3^ ER, Emergency; ^4^ OPC, Outpatient Clinic.

**Table 2:** Comparison of clinical characteristics of adult and pediatric ICU patients with *S. maltophilia* infections.

Characteristic	Adult Patients, >18 Y (*n* = 140)	Pediatric Patients, ≤18 Y (*n* = 69)	*p* Value
Mortality, *n* (%)	72 (51.4)	21 (30.3)	0.0049 **
Clinical presentation, *n* (%)			
Fever	60 (42.9)	18 (26.1)	0.0224 *
Septicemia	38 (27.1)	10 (14.5)	0.0780
Septic shock	52 (37. 1)	10 (14.5)	0.0007 ***
Bacteremia	40 (28.0)	18 (27.3)	>0.9999
GIT ^1^ symptoms	56 (40.0)	21 (30.4)	0.2226
WI ^2^	28 (20. 0)	10 (14.5)	0.4458
UTI ^3^	41 (29.3)	7 (10.1)	0.0016 **
Respiratory symptoms	99 (70. 7)	37 (53.6)	0.0203 *
Co-infection	81 (57.9)	25 (36.2)	0.0050 **
Underlying disease, *n* (%)			
Diabetes mellitus	66 (47.1)	12 (17.4)	<0.0001 ****
Hypertension	60 (42.9)	13 (18.9)	0.0006 ***
Malignancy	23 (16.4)	5 (7.3)	0.0841
Kidney illnesses	41 (29.3)	11 (16.0)	0.0414 *
Risk factors, *n* (%)			
Mechanical ventilation	132 (94.3)	49 (71.0)	<0.0001 ****
Recent antibiotic exposure	25 (17.7)	4 (5.8)	0.0189 *
Dialysis	20 (14.3)	5 (7.3)	0.1763

Note: Univariant analysis (Fisher’s exact test; *p* ≤ 0.05). The statistical significance differences between adult and pediatric patients are indicated by a (*) symbol and the number of * represent the strength of the significance difference. Data are presented as number of patients (*n*) with the corresponding percentage in parentheses (%). ^1^ GIT, Gastrointestinal Tract; ^2^ WI, Wound Infection; ^3^ UTI, Urinary Tract Infection.

**Table 3:** Univariate analysis for factors associated with mortality of ICU patients with *Stenotrophomonas maltophilia* infections.

Factor	Outcome		
	Dead, *n* = 93	Resolved, *n* = 116	*p* Value
Age above 18 y, *n* (%)			
Yes	72 (77.4)	68 (58.6)	0.0049 **
No	21 (22.6)	48 (41.4)	
Positive respiratory culture, *n* (%)			
Yes	81 (87.1)	91 (78.5)	0.1440
No	12 (12. 9)	25 (21.6)	
Septicemia, *n* (%)			
Yes	28 (30.1)	20 (17.2)	0.0321 *
No	65 (69.9)	96 (82.8)	
Septic shock, *n* (%)			
Yes	42 (45.2)	20 (17.2)	<0.0001 ****
No	51 (54.8)	96 (82.8)	
Malignancy, *n* (%)			
Yes	17 (18.3)	11 (9.5)	0.0694
No	76 (81.7)	105 (90.5)	
Prior use of carbapenem or other antimicrobials in the past 30 days, *n* (%)			
Yes	17 (18.3)	12 (10.3)	0.1106
No	76 (81.7)	104 (89.7)	
Mechanical ventilation, *n* (%)			
Yes	85 (91.4)	96 (82.8)	0.1009
No	8 (8.6)	20 (17.2)	

Note: Univariant analysis (Fisher’s exact test; *p* ≤ 0.05). The statistical significance differences between dead and resolved patients are indicated by a (*) symbol and the number of * represent the strength of significance difference. Data are presented as number of patients (*n*) with the corresponding percentage in parentheses (%).

**Table 4:** Risk factors and relative risk of mortality among ICU patients with *S. maltophilia* respiratory infections.

Variable	RR	CI 95%	*p* Value
Antibiotic pre-exposure	1.748	0.817 to 3.765	0.1818
Mechanical ventilation	1.138	1.020 to 1.290	0.0340 *
Co-infection	1.264	0.934 to 1.718	0.1690
Bacteremia	1.123	0.677 to 1.862	0.7272
Age above 18 y	1.264	1.036 to 1.557	0.0310 *

Total *S. maltophilia* respiratory positive culture (*n* = 172). The statistical significance, *p* ≤ 0.05 indicated by a (*).

**Table 5:** Comparison between ICU and Non-ICU patients with *S. maltophilia* infections.

Characteristic	ICU Patients, *n* = 209	Non-ICU Patients, *n* =184	*p* Value
**Year of 2019**	***n* = 68**	***n* = 62**	
Source of sample			
Respiratory	56 (82.4)	24 (38.7)	<0.0001 ****
Blood	5 (7.4)	11 (17.7)	0.1075
Urine	4 (5. 9)	15 (24.2)	0.0052 **
Miscellaneous	3 (4.4)	12 19.4)	0.0116 *
**Year of 2020**	***n* = 68**	***n* = 61**	
Source of sample			
Respiratory	58 (85.3)	31 (34.8)	<0.0001 ****
Blood	6 (8.8)	17 (27.9)	0.0057 **
Urine	0 (0)	9 (14.8)	0.0008 ***
Miscellaneous	4 (5.9)	4 (6.56)	>0.9999
**Year of 2021**	***n* = 73**	***n* = 61**	
Source of sample			
Respiratory	59 (80.8)	29 (47.5)	<0.0001 ****
Blood	10 (13.7)	19 (31.2)	0.0200 *
Urine	2 (2.7)	5 (8.2)	0.2448
Miscellaneous	2 (2.7)	8 (13.1)	0.0429 *

Note: Univariant analysis (Fisher’s exact test; *p* ≤ 0.05). The statistical significance differences between ICU and Non-ICU patients during the study period are indicated by a (*) symbol and the number of * represent the strength of significance difference. Data are presented as number of patients (*n*) with the corresponding percentage in parentheses (%).

## Data Availability

Data available in the KFMC institute data system and could be available for public upon special request.
